# 1-Hydroxy-8-methoxy-anthraquinon Reverses Cisplatin Resistance by Inhibiting 6PGD in Cancer Cells

**DOI:** 10.1515/biol-2019-0051

**Published:** 2019-11-15

**Authors:** Huamin Zhang, Haowei Zhang, Sihui Wang, Zhihai Ni, Tiejun Wang

**Affiliations:** 1 Department of Radiotherapy, The Second Hospital of Jilin University, Changchun, 130041, Jilin, China; 2 The Department of Clinical Laboratory, Xinhua Hospital Affiliated to Dalian University, Dalian 116021, China; 3 Department of Radiotherapy, The Second Hospital of Jilin University, Changchun, 130111, Jilin, China

**Keywords:** 6PGD, cisplatin, drug resistance, 1-Hydroxy-8-methoxy-anthraquinon, S3, combination therapy

## Abstract

Targeting 6-phosphogluconate dehydrogenase (6PGD) can inhibit cancer cell proliferation and tumor growth. However, the relationship between 6PGD and cisplatin resistance still needs further study. Cisplatin-sensitive and cisplatin-resistant ovarian cancer OV2008 and C13* lines and lung cancer A549 and A549DDP lines were treated with different concentrations of cisplatin and cell viability was evaluated. We also compared the growth rates and the cell cycle distributions between cisplatin-sensitive and cisplatin-resistant cells. The expression level of 6PGD was detected by immunoblotting. The Chou-Talalay method was used to evaluate the effect of a combination treatment using cisplatin and the small molecule inhibitor 1-Hydroxy-8-methoxy-anthraquinon (S3) that targets 6PGD. The cisplatin-resistant ovarian and lung cancer cell lines grew faster than the cisplatin- sensitive cell lines, with more cells in S and G2 phases in cisplatin-resistant cell lines. The expression level of 6PGD in cisplatin-resistant cell lines was significantly increased compared with cisplatin-sensitive cell lines. Furthermore, treatment of cells with the S3 small molecule inhibitor of 6PGD together with cisplatin could overcome cisplatin resistance. The expression level of 6PGD in cisplatin-resistant cells lines was significantly upregulated, and the resistance to cisplatin of drug-resistant cells lines could be overcome when treated with the small molecule inhibitor S3 that specifically targets 6PGD.

## 1 Introduction

Cisplatin (DDP) is a platinum-based first-line anti-cancer drug with wide applications. Presently, cisplatin is mainly used for chemotherapy of solid tumors in the clinic [1], including brain, lung, ovarian, testicular, cervical, and bladder cancers [2,3]. As a small-molecule platinum compound, cisplatin functions by first entering the cell through membrane proteins, such as copper transporter on the cell membrane. Cisplatin is composed of two Cl^-^ and two NH^3^ ions, and as the Cl^-^ concentration in the cell is relatively low, the H_2_O molecules in the cell replace the Cl^-^ ions in cisplatin, changing them into positively charged ions. Cisplatin can then bind to negatively charged molecules in the cell, including proteins, RNA and DNA. Cisplatin mainly forms cytotoxic adducts with DNA dinucleotides, causing intrastrand or interstrand DNA crosslinks; this blocks the replication and transcription processes in the cell, which halts normal cell division and leads to cell apoptosis [4].

Despite the extensive use of cisplatin in treating cancers, many patients with tumors develop cisplatin resistance at an earlier stage or after several courses of therapy. In addition, cisplatin has side effects and shows toxicity, thus seriously limiting its clinical applications. Therefore, elucidating the mechanism of cisplatin resistance in tumors has great clinical benefit [5,6].

Increasing evidence has indicated that abnormalities of cellular energy are associated with the occurrence of drug resistance in tumor cells during the course of chemotherapy [7,8]. However, the specific molecular mechanisms underlying this relationship are not fully understood. Numerous studies have reported that the effects of chemotherapy can be improved by regulating the metabolic processes in tumor cells, to some extent overcoming the resistance to anti-tumor drugs[9]. For instance, drug targeting or knocking down glucose transporter, hexokinase, pyruvate kinase M2, lactate dehydrogenase A and glucose-6-phosphate dehydrogenase (G6PD) can affect the metabolism in tumor cells and drug resistance of tumors [10,11].

The pentose phosphate pathway (PPP) is the main source of synthesis for NADPH. The three key enzymes of PPP include G6PD, 6-phosphogluconolactonase (PGLS) and 6-phosphogluconate dehydrogenase (6PGD). The third enzyme in the PPP is 6PGD, which converts 6-phosphogluconate into ribulose-5-phosphate. Additionally, 6PGD reduces NADP^+^ to NADPH and participates in the synthesis of lipids and nucleic acids. Studies show that 6PGD expression is markedly upregulated in various tumors, including colon cancer, cervical intraepithelial neoplasia, thyroid tumor and lung cancer and ovarian cancer [12, 13, 14, 15]. Activation of 6PGD is of crucial importance for the PPP and tumor growth in many tumors. Lin et al. developed a small molecule inhibitor of 6PGD, Physcion, which specifically inhibited the proliferation of leukemia cells without affecting the survival of normal somatic cells [16]. The small molecule inhibitor 1-Hydroxy-8-methoxy-anthraquinon (S3) is a derivative of Physcion and shows the same functions in inhibiting cancer cell proliferation and tumor growth. The most important limitation of our study is that we do not have in vivo results to verify the cellular level conclusions. In future research, we will continue to explore this question in animals.

Herein, we investigated whether specific inhibition of 6PGD by the small molecule inhibitor S3 can reverse cisplatin resistance in cancer cells.

## 2 Materials and methods

### 2.1 Experimental materials

Human ovarian cancer cell lines OV2008 and C13* were kindly provided by Dr. Benjamin K. Tsang from University of Ottawa, USA. Human lung cancer cell lines A549 and A549DDP were kindly provided by Professor Zhi Shi from Jinan University, China. All cell lines were subjected to cell identification.

β-actin and 6PGD antibodies were purchased from Santa Cruz Biotechnology (Santa Cruz, CA, USA). Goat anti-rabbit and goat anti-mouse secondary antibodies were purchased from Sanjian Biotech Co., Ltd. (Tianjin, China). Fetal bovine serum was purchased from Gibco Life Technologies (Carlsbad, CA, USA). RPMI 1640, trypsin and the small molecule compound S3 were purchased from Sigma-Aldrich LLC (Sigma-Aldrich, Germany).

### 2.2 Experimental instruments

The following instruments were used in this study: a SDS-PAGE (sodium dodecyl sulfate-polyacrylamide gel electrophoresis) system (EPS 300; Tianneng Technology Co., Ltd., Shanghai, China); membrane transfer system (EPS 300; Tianneng Technology Co., Ltd.); gel imaging system (Universal Hood III; Bio-Rad, Hercules, CA, USA); and multi-function microplate reader (Multiskan FC; Thermo Scientific, USA).

### 2.3 Cell Culture

Cisplatin resistance in the cisplatin-resistant cell line A549DDP was maintained by culturing cells with a low concentration of cisplatin; the ovarian cancer cell line C13* is naturally resistant to cisplatin. All cell lines were cultured in an incubator at 37℃ with 5% CO_2_. Cells were passaged or cryopreserved based on their proliferation rate.

### 2.4 Cell Proliferation Assay

Cell proliferation assays were performed by seeding 5×10^4^ cells in 6-well plates. Relative cell proliferation was determined bycell counting at 3 days after being seeded and the percentage cell proliferation of the control. Cell growth was determined by cell numbers recorded at 0, 1, 2, 3, and 4 days after being seeded.

### 2.5 Flow cytometry analysis of cell cycle

OV2008, C13*, A549 and A549DDP cells were seeded in six-well plates respectively and cultured for 24 h. For cell cycle analysis, treated cells were fixed using 70% ethanol for 24 h followed by propidium iodide staining. Cells were analyzed using FACScantoII cytometers (BD Biosciences; SanJose, CA, USA). All analyses were repeated in triplicate.

### 2.6 Intracellular 6PGD enzyme activity assay

Total protein was extracted from the cells using RIPA Lysis and Extraction Buffer (ThermoFisher, China). The protein in cell lysates was quantified and samples were adjusted to the same protein concentration. Because 6PGD catalyzes the reduction of NADP^+^ to NADPH, 6PGD enzyme activity is determined through the accumulation of NADPH produced. An aliquot of cell lysate (10 μg protein) was added into the reaction buffer (0.1 mmol·L^-1^ MgCl_2_, 50 mmol·L^-1^ Tris pH 8.1, and 0.2 mmol·L^-1^ 6-phosphogluconate), followed by the addition of NADP^+^ with a final concentration of 0.1 mmol·L^- 1^. Finally, an enzyme kinetics program was set in the multifunctional microplate reader (one measurement every 20 s for 10 min). The absorbance was measured at a wavelength of 340 nm.

### 2.7 Western blot assay

When cells reached 80%–90% confluence, cells were harvested and lysed using RIPA Lysis buffer, and total protein was quantified. Samples were separated by SDS-PAGE and then transferred to a polyvinylidene fluoride membrane. The membrane was blocked with 5% skimmed milk powder for 2 h and then incubated with primary antibodies (β-actin 1:2000 catalog number: sc-47778; 6PGD 1:1000, catalog number: sc-398977) on a shaker at 4℃ overnight. Membranes were then incubated with the corresponding secondary antibody (anti-mouse 1:1000 or anti-rabbit 1:1000) for 2 h. The membranes were then incubated with a chemiluminescent solution for 1 min and exposed with a Bio-Rad gel imaging system.

### 2.8 MTT assay

Cells were plated in 96-well plates at a density of 4000 cells per well in a volume of 200 μL per well. After incubation overnight, cells were treated with drugs for 24 h (for EC50 assay, S3 0-40 μg/mL) and 2 d (for cell proliferation assay), respectively. Subsequently, 20 μL of MTT (thiazolyl blue tetrazolium bromide) solution (5 mg/mL in PBS, pH 7.4) was added to each well and cells were incubated for another 4 h. The supernatant was carefully aspirated from the wells and 150 μL of DMSO (dimethyl sulfoxide) was added into each well, followed by oscillation for 10 min. The absorbance in each well was measured and recorded at a wavelength of 570 nm. Cell growth curves were drawn with drug concentration as the abscissa and the absorbance as the ordinate.

### 2.9 Statistical analysis

All experiments were performed with three replicates and data were subjected to an analysis of variance by t-test using GraphPad Prism 5.0 (GraphPad Software, Inc., San Diego, CA, USA). A *P* value of less than 0.05 was considered to indicate statistical significance.

## 3 Results

### 3.1 6PGD expression is upregulated in cisplatin-resistant cell lines

**Figure 1:**
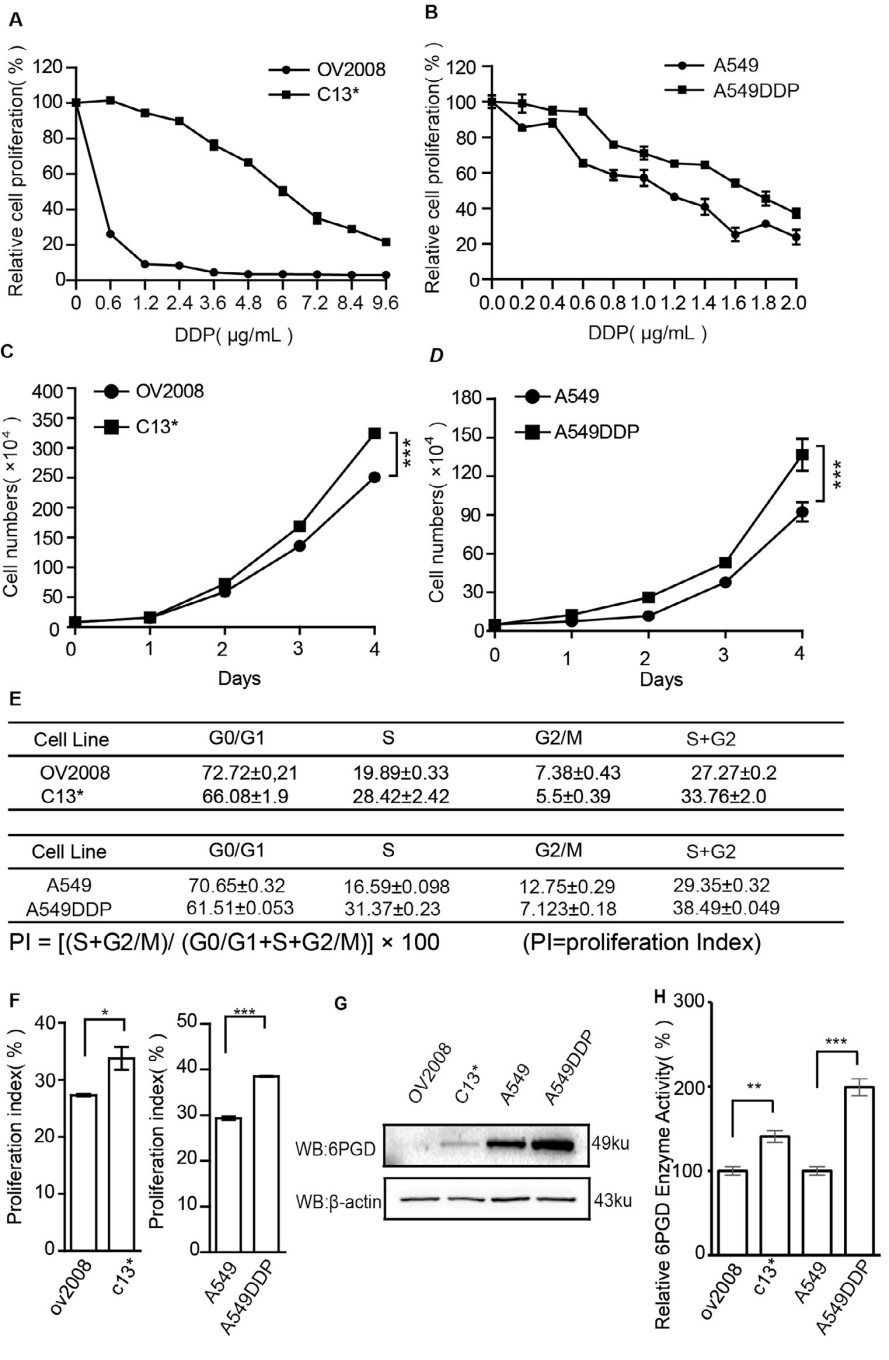
**6PGD expression is upregulated in cisplatin resistant cells**. A and B: Cisplatin-resistant cell lines C13* and A549DDP and the cisplatin-sensitive parental cell lines OV2008 and A549 were treated with different concentrations of cisplatin and cell proliferation rates were determined by CCK-8 assays. C and D: Proliferation rates of cisplatin-resistant cell lines C13* and A549DDP and the cisplatin-sensitive parental cell lines OV2008 and A549 were determined by cell counting. E and F: Cell cycle distributions of cisplatin-resistant cell lines C13* and A549DDP and the cisplatin-sensitive parental cell lines OV2008 and A549 were determined by flow cytometry. G: Western blot of 6PGD in cisplatin-resistant cell lines C13* and A549DDP and the cisplatin-sensitive parental cell lines OV2008 and A549. Error bars represent mean values ± SD from three replicates of each sample. .H: Enzyme activity assay of 6PGD enzyme activity in OV2008, C13*, A549, and A549DDP cells. *P< 0.05; **P< 0.01; ***P< 0.001

To explore the mechanism of cisplatin resistance in lung and ovarian cancers, we used cisplatin-sensitive and cisplatin-resistant ovarian cancer cell lines (OV2008 and C13*, respectively) and lung cancer cell lines (A549 and A549DDP, respectively). We confirmed that OV2008 and A549 cells were more sensitive to cisplatin compared with their counterparts, C13* and A549DDP cells (figure 1A, B) We also found that the cisplatin-resistant cell lines had a higher cell proliferation rate than the cisplatin-sensitive cell lines (figure 1C, D) Cell cycle analysis revealed more cells in S and G2 phases in cisplatin-resistant cell lines (figure 1E, F), which confirm the cell proliferation results.

Several lines of evidence suggest that cancer cells upregulate the oxidative PPP to support cell growth and survival [18]. However, the precise contribution of PPP in drug resistance has not yet been studied. Based on our results showing increased proliferation in cisplatin-resistance cell lines, we speculated that the key enzymes of PPP may be upregulated to a certain degree. The results showed that 6PGD expression was markedly upregulated in cisplatin-resistant cell lines compared with cisplatin-sensitive lines (figure 1G) And then we compared enzyme activity between cisplatin-resistantand cisplatin-sensitive cells. These results showed that 6PGDe nzyme activity was upregulated in cisplatin-resistant C13 and A549DDP cells (figure 1H) Our results suggest that cisplatin-resistant cell lines may accelerate cell proliferation by upregulating 6PGD, thus resulting in cisplatin-resistance.

### 3.2 S3 can specifically inhibit 6PGD enzyme activity

**Figure 2:**
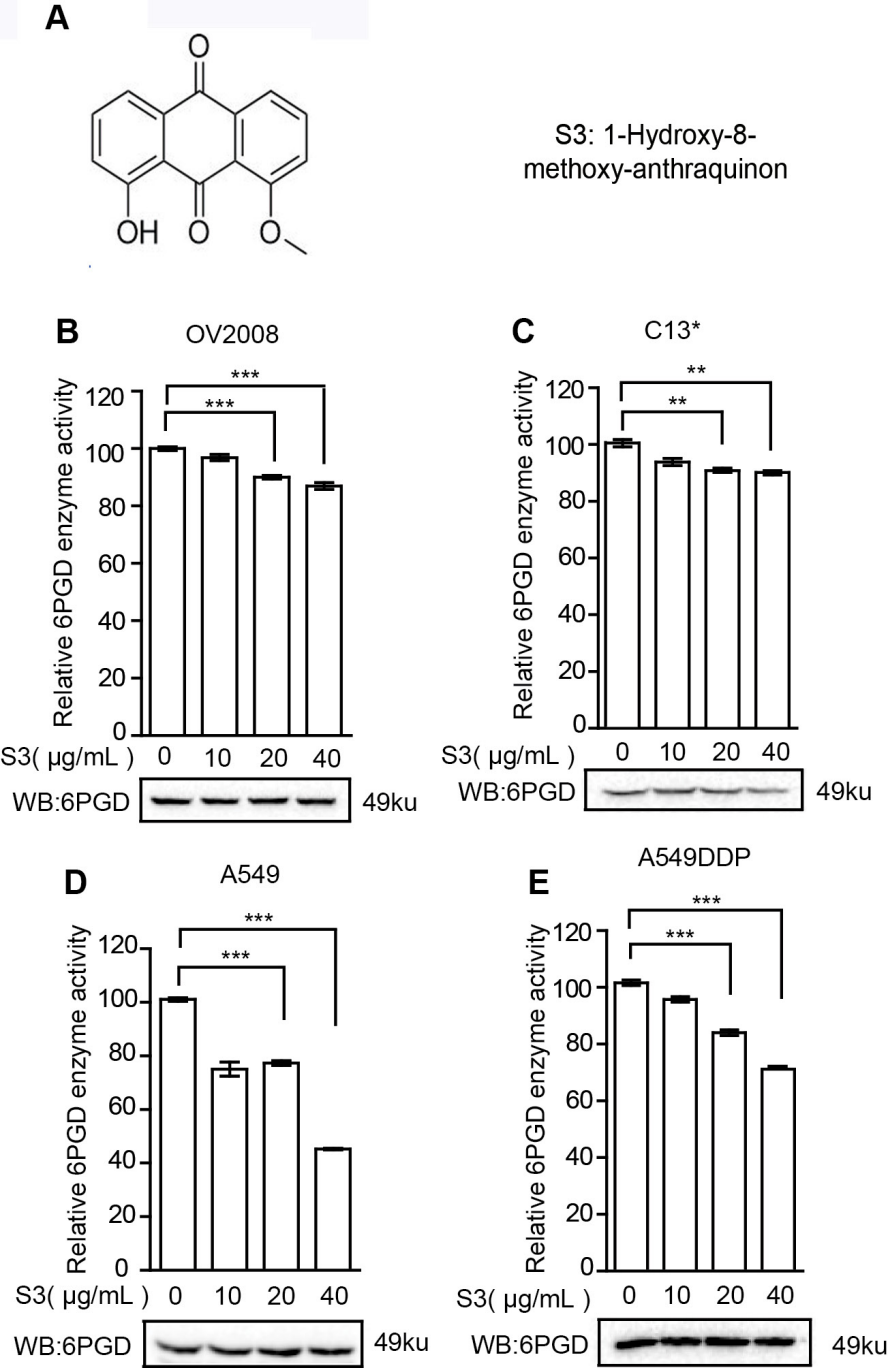
**S3 specifically inhibits 6PGD enzyme activity without affecting 6PGD protein expression**. A: Structure of the small molecule inhibitor S3. B–D: 6PGD enzyme activity (upper) and 6PGD protein expression (lower) in OV2008 cells (B), C13* cells (C), A549 cells (D) and A549DDP cells. E: in the presence of increasing concentrations of S3 were determined by enzyme activity assay and western blot, respectively. Error bars represent mean values ± SD from three replicates of each sample. *P< 0.05; **P< 0.01; ***P< 0.001.

To explore the role of 6PGD in tumor drug resistance and the potential for 6PGD as a clinical target, we used the S3 small molecule inhibitor of 6PGD, which can decrease 6PGD enzyme activity without affecting the expression level of 6PGD [16]. We treated the ovarian and lung cancer cell lines with 10, 20 or 40 μg/mL S3 for 24 h. Then the cell lysates were collected and the total protein of 30 μg per well was used for western blot detection. The results showed that 6PGD enzyme activity significantly decreased after S3 treatment, while 6PGD protein expression levels did not significantly change (figure 2A–E) This indicates that S3 can target 6PGD to inhibit its enzyme activity without affecting its protein level.

### 3.3 S3 attenuates cell proliferation

**Figure 3:**
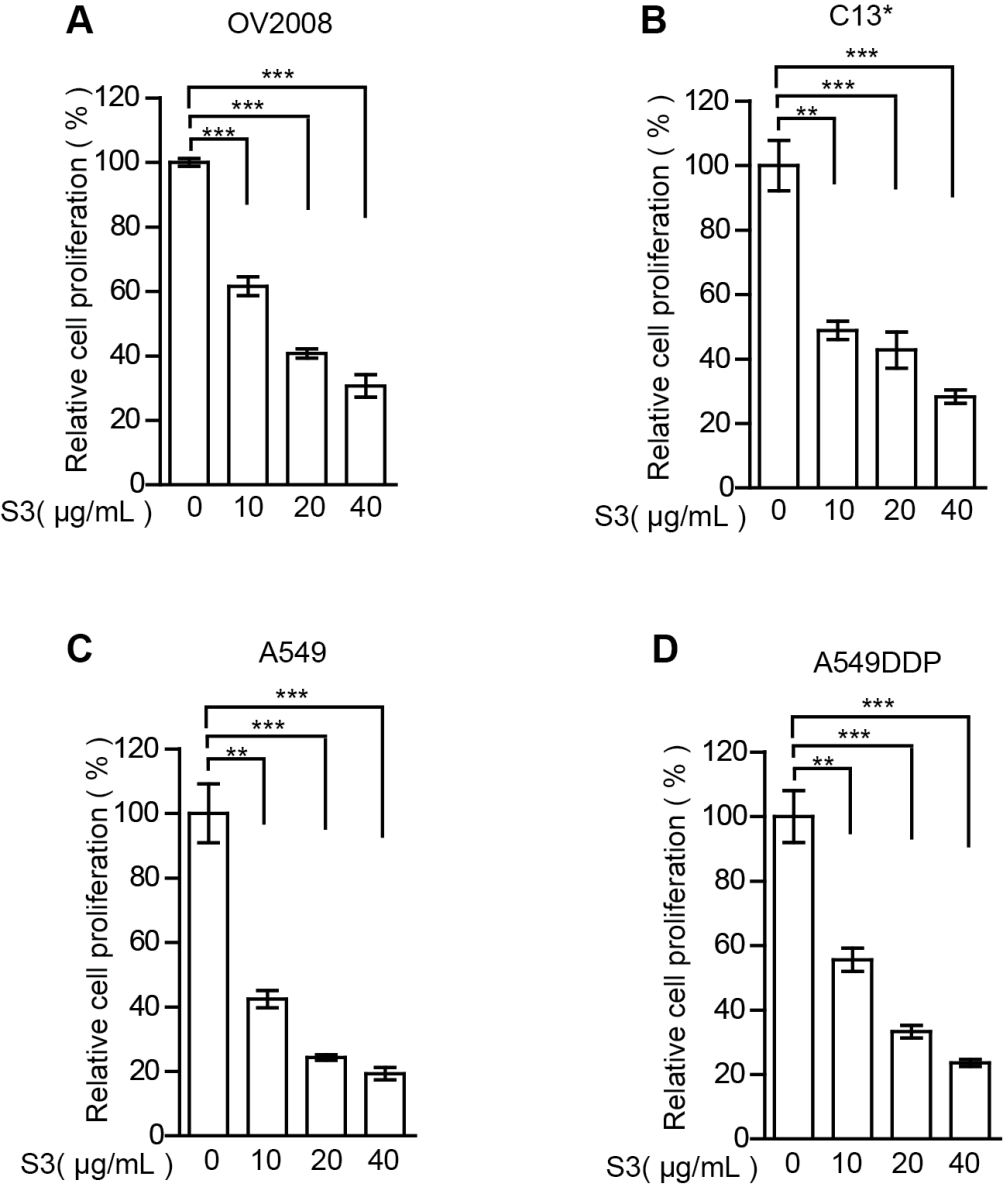
**S3 attenuates cell proliferation** A–D: Cell proliferation rates of OV2008 cells (A), C13* cells (B), A549 cells (C) and A549DDP cells (D) in the presence of increasing concentrations of S3 were determined by cell counting. Error bars represent mean values ± SD from three replicates of each sample. *P< 0.05; **P< 0.01; ***P< 0.001.

Activation of 6PGD is of crucial importance for the PPP and tumor growth in many tumors. Our results showed that 6PGD is significantly upregulated in cisplatin-resistant cell lines and that the small molecule inhibitor S3 can specifically target 6PGD. We next treated the cells with various concentrations of S3 for 3 days and evaluated cell proliferation. The results showed that S3 significantly attenuated the cell proliferation (figure 3A–D) At last, we valuated for cytotoxic activity against OV2008, C13*, A549 and A549DDP cells, the median effect concentration (EC50) values were 16.33, 11.17, 10.02 and 8.657 μg/mL, respectively (Graphpad Prism 5).

### 3.4 Combined treatment with S3 and cisplatin greatly increases cell mortality with a synergistic effect

**Figure 4:**
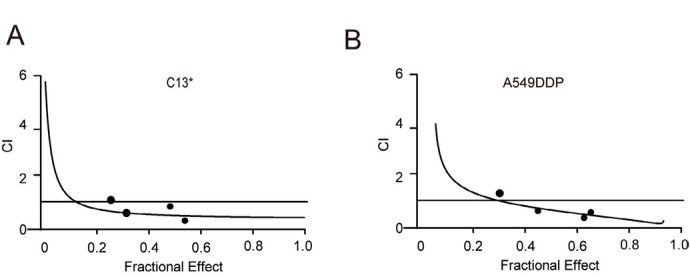
**Inhibition of 6PGD can reverse cisplatin resistance in cancer cell lines**. A–B: A549DDP and C13* cells were treated with a serial constant-ratio combining S3 and cisplatin for 48 h. The cell viability-based synergistic effect of S3 and cisplatin was analyzed according to approach described by Chou and Talalay. A combination index (CI) less than 1 indicates a synergetic effect.

The Chou-Talalay method for drug combination is based on the median effect equation. In principle, the combined use of drugs is bound to induce continuous changes in the pharmacokinetic parameters of the drugs per se. These parameter changes can reflect the interactions between drugs from qualitative and quantitative perspectives. Finally, the combination index (CI) can quantitatively describe the additive (CI =1), synergetic (CI <1) or antagonistic (CI >1) effects of drug combination [17]. The combined treatment of S3 and cisplatin showed a CI <1 for the cisplatin-resistant cell lines C13* and A549DDP, indicating a synergistic effect of this drug combination (figure 4A, B) This indicates that cell mortality was greatly increased in the cisplatin-resistant cell lines C13* and A549DDP after combined treatment with S3 and cisplatin, with a synergistic effect.

## 4 Discussion

Chemotherapy is one of the most important strategies for the treatment of malignant tumors. However, tumor cells can show resistance to chemotherapeutic drugs, which often leads to failure of chemotherapy [18]. For example, cisplatin has been extensively used to treat various solid tumors, but cisplatin resistance occurs in the early stage of therapy for many tumors. Tumor cell resistance can be divided into two classes, intrinsic (present without contact with drugs) and acquired (generated after contact with drugs). In tumors that show drug resistance, one or more drugs are usually combined to achieve a better therapeutic effect [19]. Compared with immunotherapy, the combined use of anti-cancer drugs enhances the therapeutic effect, as it typically targets critical pathways with synergistic or additive effects. This method potentially reduces tumor drug resistance while bringing therapeutic anticancer benefits, for example, attenuating tumor growth and metastasis potential, blocking cell proliferation, reducing cancer stem cell populations and inducing cell apoptosis [16].

Activation of 6PGD has great implications for the PPP and tumor growth in various tumors. 6PGD can inhibit LKB1-AMPK signaling and link the PPP with the lipid synthesis pathway, accelerating tumor growth. Thus, 6PGD can be used as a potential anti-cancer target[16]. S3 is a derivative of Physcion, which is a small molecule compound extracted from emodin. Physcion can specifically target 6PGD and has been shown to effectively block the proliferation of tumor cells with no off-target effect and low hepatocellular toxicity [20].

In this study, we found a higher cell proliferation rate in cisplatin-resistant cell lines compared with cisplatin-sensitive cell lines. We also demonstrated the upregulation of 6PGD, an enzyme in the PPP, in cisplatin-resistant cell lines compared with cisplatin-sensitive lines. We found that S3 specifically targeted 6PGD to inhibit its enzyme activity and attenuate cell proliferation in ovarian and lung cancer cells. A combination treatment of the small molecule inhibitor S3 with cisplatin significantly improved cisplatin sensitivity and increased cell mortality in cisplatin-resistant cell lines, with a synergistic effect. Taken together, these results suggest that cisplatin resistance in cisplatin-resistant cell lines can be overcome by specific inhibition of 6PGD enzyme activity using the small molecule inhibitor S3.

The process of developing new anti-cancer drugs is highly expensive and time consuming. Therefore, current cancer treatment strategies often involve combining one or several other drugs, which has reduced the overall cost of combined combination therapy studies and simultaneously increased the cost-effectiveness of therapy. Additionally, the combination of drugs with other therapeutic agents has shown promising results with respect to reducing the tumor burden[20]. Therefore, it is necessary to explore the molecular mechanisms of drug resistance in tumors to help develop strategies for overcoming chemotherapy resistance in the future.
